# Supplementary material for: Farming Practices, Biosecurity Gaps, and Genetic Insights into African Swine Fever Virus in the Iringa and Ruvuma Regions of Tanzania
Source: Animals (Basel). 2025 Mar 31;15(7):1007. doi: 10.3390/ani15071007 (PMC11987749; doi:10.3390/ani15071007)
Supplement: Supplementary file 1 [file animals-15-01007-s001.zip › animals-3454934-supplementary.pdf]

Supplementary data S1: questionnaire for farmers visited.

1. **Respondent Information:**

Name and date:
Work Affiliation:
Email address:

2. Farm and Sample Details
   Sample ID: ________________________
   Farm Locality: ________________________
   Department: ________________________ Province: ________________________
   Latitude (decimal format, e.g., -1.23456): ________________________________
   Longitude (decimal format, e.g., -1.23456): ________________________________
   Species (specific pig breed, if applicable): ____________________ Sex: __ Age: ________
   Sample Type Collected (e.g., blood, tissue): ____________________

3. Describe the Disease Progression (e.g., onset of symptoms, severity):
   ____
   ____

4. A. Type of Farming System
   Free-ranging
   Semi-confined
   Confined
   other (please specify): ________________________

   B. Potential Interaction with Wildlife:
   Yes
   No

   C. If there is a potential interaction with Wildlife, please indicate the Type of Wildlife:
   Warthogs (Phacochoerus africanus)
   Bushpigs (Potamochoerus larvatus)
   Giant Forest Hogs (Hylochoerus meinertzhageni)
   other (please specify): ________________________

5. Housing Conditions Assessment for Pig Environments

   For an improved assessment of the housing conditions, detailed information about the pig's environment is requested. Please provide input based on the Housing Conditions Scoring Rubric aligned with WOAH (World Organisation for Animal Health) Welfare Standards for Pigs.

   Assign a score for each category based on the following criteria, where:

   - 1 reflects non-compliance with WOAH standards,
   - 2 indicates partial compliance with significant room for improvement,
   - 3 indicates basic compliance with WOAH standards,
   - 4 indicates good compliance with only minor deficiencies noted,
   - 5 indicates full compliance with WOAH standards and possibly exceeding requirements.

Space Allowance:
- Evaluate the space available per pig against WOAH guidelines.
- Score (1-5): _____

Temperature and Environmental Control:
- Evaluate how well the housing manages temperature extremes and provides adequate weather protection.
- Score (1-5): _____

Air Quality:
- Assess ventilation systems and measures to maintain good air quality.
- Score (1-5): _____

Feed and Water:
- Evaluate the accessibility and quality of feed and water provisions.
- Score (1-5): _____

Behavioral Enrichment:
- Assess whether pigs have access to materials and environments that allow for natural behaviors.
- Score (1-5): _____

Health Management:
- Evaluate disease control measures, biosecurity protocols, and veterinary care adequacy.
- Score (1-5): _____

Safety and Emergencies:
- Assess the measures in place for dealing with emergencies and protecting pigs from harm and injury.
- Score (1-5): _____

Comments:
- Provide any additional comments or observations regarding the housing conditions.

Visual Evidence:
- Include photographs or video evidence of the housing conditions where possible.

6. A. Purpose of Sample Collection:
   Surveillance
   Outbreak investigation, if outbreak go to question B
   other (please specify): ________________________

   B. ASFV Outbreak Information
   Morbidity (Number of affected animals): ________________________
   Mortality (Number of deceased animals): ________________________
   Date of the Outbreak (DD/MM/YYYY): ________________________
   Number of Animals on Farm Before Outbreak: ________________________

7. ASFV infection status based on PCR analysis
   Positive
   Negative

Suspected

8. Were there any signs at the time of collection?
   Yes (Please specify): ________________________
   No

9. Describe any observed immune responses (e.g., recovery, resistance to subsequent infections - For example, after recovering from ASFV, did the animal show an increased ability to fend off the same illness if exposed again?): ____
   ____

10. Were there any co-infections identified?
    Yes (Please specify): ________________________
    No

11. Please provide any additional comments or suggestions related to the questionnaire: ____
    ____
    ____
    ____
    ____

Supplementary data S2: ASF positive and suspected samples sent to APHL

Sample ID	Origin	Sample Location
Tan_ASF2024_01	Ruvuma Region	Songea_ Subira ward
Tan_ASF2024_02		
Tan_ASF2024_03		
Tan_ASF2024_04		
Tan_ASF2024_05		
Tan_ASF2024_06		
Tan_ASF2024_07		
Tan_ASF2024_08		
Tan_ASF2024_09		
Tan_ASF2024_10		
Tan_ASF2024_11		
Tan_ASF2024_12		
Tan_ASF2024_13		Songea_ Mateka ward
Tan_ASF2024_14		Songea_ Msamala ward
Tan_ASF2024_15		
Tan_ASF2024_16		
Tan_ASF2024_17		Songea_ Lizabon ward
Tan_ASF2024_18		
Tan_ASF2024_19		
Tan_ASF2024_20		
Tan_ASF2024_21		Songea_ Msamala ward
Tan_ASF2024_22		
Tan_ASF2024_23		
Tan_ASF2024_24		
Tan_ASF2024_25		
Tan_ASF2024_26	Iringa Region	Iringa Dc _ Pawaga
Tan_ASF2024_27		
Tan_ASF2024_28		Iringa Mc_ Ipogoro
Tan_ASF2024_29		
